# Shame in decision making under risk conditions: Understanding the effect of transparency

**DOI:** 10.1371/journal.pone.0191990

**Published:** 2018-02-14

**Authors:** Tomas Bonavia, Josué Brox-Ponce

**Affiliations:** Department of Social Psychology, University of Valencia, Valencia, Spain; Centre national de la recherche scientifique, FRANCE

## Abstract

The role played by the emotion of shame in the area of decision-making in situations of risk has hardly been studied. In this article, we show how the socio-moral emotions and the anticipated feeling of shame associated with different options can determine our decisions, even overriding the cognitive choice tendency proposed by the certainty effect. To do so, we carried out an experiment with university students as participants, dividing them into four experimental conditions. Our findings suggest that people avoid making unethical decisions, both when these decisions are made public to others and when they remain in the private sphere. This result seems to indicate that the main factor in not making unethical decisions is related to the need to avoid transgressing an internal moral standard of behavior, and that the role of transparency is less relevant than expected. However, we propose that, although the effect of transparency is limited in reducing unethical economic decisions, it should continue to be taken into account in theoretical models that address the reasons people behave unethically.

## Introduction

The field of decision-making in risk situations has been dominated by two important theories: Expected Utility Theory [1] and Prospect Theory [2]. According to Expected Utility Theory, people rationally choose those results that present greater expected utility. In the case of Prospect Theory, the decisions depend on the point of reference or status quo, so that better results than the point of reference are considered gains, and worse results are considered losses [3]. This theory also proposes the existence of a pattern of four attitudes toward risk: risk aversion for high probability gains, risk seeking for high probability losses, risk seeking for low probability gains, and risk aversion for low probability losses [4]. Underestimating the value of probable gains compared to sure gains is called the “certainty effect”, whereas overestimating the value of probable losses compared to sure losses is known as the “reflection effect” [2]. It is true that Prospect Theory introduced a necessary shift in the comprehension of decision-making, as until then it had been described through rational choice models. However, under certain conditions, its theoretical framework appears to be insufficient to explain some decisions affected by our emotions. This study will highlight the need to formulate a new theoretical model that consider the role of socio-moral emotions in decision-making, as well as the importance of distinguishing the different effects of various socio-moral emotions on decision-making in risk situations. Specifically, the role played by the anticipated emotion of shame will be clarified, as this socio-moral emotion has received less attention in the scientific literature [5]. Thus, the study will show how and to what extent the anticipated emotion of shame can reduce the incidence of economic decisions that involve moral transgressions. We think the explanation of this mechanism can help to clarify the influence of transparency measures on non-ethical behaviors.

### Affect and emotion in decision-making under risk conditions

Although Prospect Theory is capable of explaining some typical deficiencies of the Expected Utility Theory, it does not take into account the implicit emotions that can be present in different choices. From this point of view, emotions play an incidental role and are not integrated in the decision-making process. Loewenstein, Weber, Hsee and Welch [6] refer to these theories as cognitive and consequentialist because they assume that people make choices by evaluating the consequences of the possible response alternatives. Therefore, they give excessive importance to the probabilistic evaluation and the possible outcomes. In an attempt to provide an alternative view of these issues, these authors suggested the “risk as feeling hypothesis”, pointing out that decisions made in risk situations are partly the result of the direct influence of feelings. From this perspective, behavior and decision-making depend on the mutual influence between the cognitive evaluation (nature and probability of outcomes) and affective information (vividness with which possible consequences can be imagined, previous experiences with the outcomes, and past conditioning). In a similar way, Slovic, Finucane, Peters and MacGregor [7] propose that mental images are marked by positive and negative emotions that guide judgement and decision-making. People would use the “affect heuristic” to affectively label stimuli they have experienced before. Damasio [8] explains this in his “somatic marker hypothesis”. He proposes that in the moment of making a judgement or decision, positive and negative emotions work as corporal markers that guide the attention toward certain future results. In other words, negative somatic markers function as an alarm signal, whereas positive somatic markers serve as action incentives.

In spite of these discoveries, very few empirical investigations have tried to integrate affective aspects within response alternatives in order to examine the effect on Prospect Theory predictions. Rottenstreich and Hsee [9] performed a series of experiments in which the participants had to choose between affect-rich and affect-poor prizes, with the purpose of proposing an affective reconstruction of the weighting function originally proposed by Kahneman and Tversky [2]. In their words: “*people were more sensitive to departures from impossibility and certainty and less sensitive to intermediate probability variations for affect-rich than for affect-poor prizes”* (p.188). In their conclusions, the authors point out the need to propose new models that consider how the affective nature of the results can influence the evaluation of the probability that a possible effect will occur. Along these lines, Bonavia [10] found that the certainty and reflection effect does not take place when the decisive stimuli carry some implicit affective value. Specifically, people preferred a probable gain and a sure loss of the affect-rich alternative, rather than a sure gain and a probable loss of an affect-poor alternative.

In this study, in order to determine the effect of emotions on decisions in risk situations, we will examine the effect of anticipated shame on the predictions of Prospect Theory. Even though many studies take into account the effect of several discrete emotions on judgement and decision-making, socio-moral emotions like shame have hardly been studied in the decision-making field [5]. Given that information about shame will be provided in the following section, first some studies will be presented that have focused on similar emotions, such as “embarrassment” or “guilt”.

Regarding embarrassment, studies have shown that feeling anxiety when facing the possibility of experiencing an embarrassing situation leads to a search for risk [11]. This conclusion was supported by Coffaro [12], who, in a series of experiments, discovered that a state of embarrassment can lead to a reduction in the perception of risk when making a decision. In both cases, the emotion of embarrassment was not related to the decision problem used to estimate the assumed risk, but rather it arose before choosing the alternatives (remembering or experiencing an embarrassing situation). However, the opposite conclusion was reached when the state of embarrassment was related to the decision problem. Goulart, Da Costa, Andrade and Santos [13] showed that when people expected their financial performance results to be published, the disposition effect increased; that is, people sold more profitable shares in order to guarantee their benefits and avoid the embarrassment of bad decisions. This conservative attitude toward anticipated embarrassment contrasts with the search for risk stemming from the embarrassment experienced in previous studies.

With regard to “guilt”, Mancini and Gangemi [14] showed that several emotional states can influence the process of reasoning and decision-making under risk in different ways. They assigned the participants in profit and loss situations and found that, by inducing a mental state of guilt, people invariably experienced risk aversion. By contrast, when people were informed that they had been victims of an unfair decision, they sought risk in both situations. These results reveal that the decisions were determined mainly by the influence of an emotional state, rather than by the *framing effect* that would be expected if Prospect Theory predictions were fulfilled.

So far, this article has presented the main theories on decision-making in the field of risk, as well as the main conclusions from research about the influence of some discrete emotions on the predictions of these theories. The following section will explain what is known about the effect of shame on judgment and decision-making.

### Shame and behavior

Shame is a self-conscious, socio-moral emotion that involves making an overall negative evaluation of the self [15–17]. Its appearance requires having previously developed certain cognitive abilities that allow one to establish a clear differentiation between the self and others, in addition to having some standards or norms about what is right and wrong [18]. A person who experience shame has the desire to avoid, escape, or hide from the situation that creates this emotion [19]. Likewise, shame is an extremely painful emotion that is usually accompanied by a shrinking sensation and feelings of impotence and uselessness [20]. Two important characteristics distinguish the emotion of shame from other socio-moral emotions such as guilt or embarrassment. First, the emotion of shame appears in situations where there is a transgression of a moral rule or the violation of some moral standards of behavior [21], whereas the emotion of embarrassment is associated with relatively innocuous violations, such as losing control over one’s body, making cognitive mistakes, or exhibiting deviations in one’s physical aspect [22]. Second, anthropology has pointed out that shame is a public emotion that stems from the disapproval of others [23, 24]. Although this conception of shame has been strongly criticized [16, 17], it cannot be denied that the public dimension provides a complementary way to understand the complexity of this emotion. Smith, Webster, Parrott and Eyre [25] showed that the emotion of shame was more linked to feelings caused by publicly exposing transgressions or incompetence than the emotion of guilt. Consequently, we can state that shame is an emotion linked to public exposure after the transgression of moral standards of behavior, although it does not always arise in these circumstances.

One of the most important theories on how shame can modify behavior is the “Sociometer Theory” [26–28]. This theory points out that people experience a strong motivation to maintain at least a few interpersonal relationships because they are sensitive to being or not being accepted by others. Through a “sociometer”, people examine how they are perceived by other people, and they are interested in not transmitting inappropriate impressions that put their social inclusion at risk. By doing so, they try to avoid a possible devaluation of their interpersonal relations, considering that it might lead to negative consequences, both physical and psychological [29]. In this regard, when people detect symptoms of a possible devaluation of their social relationships, they experience a decrease in their self-esteem levels, which at the same time triggers the motivation to be accepted by others again. In addition, the perception of this devaluation not only produces effects on self-esteem levels, but it also produces negative social emotions such as shame [30]. From this perspective, people feel ashamed when they believe that their behavior or personal traits weaken their relationships with other people. Therefore, we think that shame can serve as a preventive emotional mechanism to avoid the expression of behaviors that deviate from the moral norms [21, 31]. This idea makes it possible to differentiate between anticipated shame and experienced shame (experienced shame and its effects are described above). Anticipated shame is thought to make us avoid certain behaviors, even when they can be beneficial, and it has less weight in decision-making than experienced shame [32]. More specifically, some authors have proposed that anticipated shame prevents us from engaging in immoral behaviors [31]. An example of this would be the existence of a negative relationship between anticipated shame and our offenses toward other people [33].

In addition, it has been suggested that socio-moral emotions such as shame can motivate people to act in an ethical way [34]. Evidence for this comes from the experiment carried out by Jacquet, Hauert, Traulsen and Milinski [35], who tested their participants on the well-known dilemma of public good game. In their experiment, the authors divided the participants into three conditions: shame, honor, and control. In the conditions of shame and honor, the most selfish and generous participants could be identified by the other members of the group, whereas in the case of the control group, the participants’ identity remained anonymous to both the participants and the experimenter. At the beginning of the experiment, all the participants received the same amount of money. Throughout 12 rounds, the participant had to decide whether to contribute $1 to a public pool or keep it in his/her private funds. The results showed that when participants ran the risk of feeling ashamed or honored by publishing their decisions, they showed greater cooperation with each other. In this line of research, Milinski, Semmann and Krambeck [36] found that by alternating the rounds in a public good game with the rounds of an indirect reciprocity game, the players maintained higher levels of cooperation than if they first played all the rounds of the public good game. This occurred because in the indirect reciprocity game, the players could be rewarded if they had maintained a good reputation in the eyes of the other players in the public good game. In another similar experiment, the same authors showed that the level of cooperation increased if people could be recognized with the same identity in both games, while it decreased when their identity changed from one game to another [37]. Together, all these experiments reveal that cooperation increases when people’s identities are kept public to the other group members. More specifically, the experiments showed that anticipated feelings of shame were able to motivate more collaborative behavior. These feelings can vary in strength depending on the psychological proximity of the people affected by our behavior, as Ghorbani, Liao, Caykoylu and Chand conclude [38]. According to their results, the closer the victims affected by the behavior are, the greater the level of shame experienced by the offender. They found that participants showed higher levels of shame when the victims of the actions were members of their reference group, followed by strangers with a specific description, and, finally, by strangers described in a general or abstract way. Having said that, we do not intend to suggest with these results that cooperation cannot be increased by factors unrelated to maintaining one’s reputation. Other experiments have shown that people do not reduce their disposition to act with reciprocity, even when the opportunities for cooperation remain anonymous [39], which means that reciprocity does not exclusively depend on the anticipation of social sanctions or conformity with the behavior of members of our group.

Moreover, from an economic perspective, behavioral models have been proposed that try to explain dishonesty by acknowledging the importance that external rewards have for people. The classical rational model takes factors into account that influence dishonest acts, focusing on the cost-benefit analysis of external rewards and punishments. From this perspective, people who intend to be dishonest estimate the amount of money they can win, the chances of being caught, and the severity of the punishment if caught [40]. Nevertheless, for Mazar, Amir and Ariely [41], the dishonesty of our acts is determined by internal considerations, such as our attention to moral standards and the possibility of reinterpreting or categorizing our acts so they do not seem wrong to us. These authors believe that two factors compete in determining the degree of (dis)honesty of an act: the benefit we obtain from cheating and the need to maintain a positive self-concept. Thus, people resolve this tension by searching for a balance between these two motivating forces. They try to obtain some financial benefit by behaving dishonestly while trying to maintain a positive view of themselves in terms of honesty. Although this theory offers a more complete vision of the rational models, it does not contemplate that our dishonesty could diminish if our decisions were made public to others. This does not mean that these models are inappropriate, but in a context where people’s decisions can be seen by others, they might find it relevant to avoid criticism and behave according to external moral standards of behavior.

So far, we have shown the importance of taking into account other approaches that integrate emotional aspects in decision-making in the area of risk, emphasizing the importance of considering the influence of discrete emotions such as shame. The experiments and previous theories suggest that the emotion of shame can be useful to favor cooperation and avoid moral transgressions. In order to further examine the positive aspects of the emotion of shame, the following section will introduce one of the political devices linked to beneficial aspects of this emotion: transparency in economic decisions.

### Transparency as a public decision

Transparency has been understood in multiple ways, and different types have even been considered based on different branches of knowledge. Generally, transparency is defined as the open flow and public access to information [42]. In the field of decision-making in risk situations, we understand a transparent decision as a public decision, that is, a decision about which information is accessible to everyone in our social environment. There is a consensus in the scientific literature about relating lack of transparency to high corruption levels, even though this is usually nuanced by saying that it plays a moderator role that depends on other factors [43]. In this regard, it has been suggested that transparency measures alone are not enough because they should be accompanied by other measures that improve people’s capacity to access and process the available information [44]. However, Florini [45] points out that transparency has brought benefits to society, such as improving the functions of financial markets and attracting investment, along with the detection, control, and modification of policies of governmental and international institutions.

Based on the above, it is important to emphasize that transparency has classically been studied from a political and macroeconomic point of view by focusing on the effects of certain transparency measures on different economic parameters. However, this article emphasizes the influence of transparency measures at a microeconomic level, focusing on people’s economic decisions that are made public. Therefore, we propose that the fact that our decisions are exposed publicly can produce anticipated feelings of shame that push us to avoid making unethical decisions, even when they provide personal economic benefits [25, 40]. Thus, we believe that making decisions transparent can increase the degree of exposure and risk of rejection by others [30]. In short, this study offers information about the key mechanisms that allow us to explain how and to what extent transparency is capable of reducing the incidence of unethical behaviors.

### Objective and hypotheses

Both the Sociometer Theory and affective approaches suggest that feelings of shame can make people more cooperative and define what behaviors should be avoided to protect their self-esteem and social image. However, it is not clear whether this latter effect can have a direct influence on the economic decisions we make and keep us from making unethical decisions that can be favorable in economic terms.

For this reason, the main objective of this study is to analyze whether there is a reduction in unethical choices when they are made public, compared to when they remain anonymous. In addition, it can be shown that the emotional charge implicit in the alternatives is capable of annulling the certainty effect predicted by Prospect Theory. Specifically, we expect to show that aversion to the unethical option is more powerful than aversion to risk in a hypothetical situation of profit, which reinforces the importance of valuing the emotional aspects in decision-making in risk situations. Therefore, we propose the following hypotheses for a situation of gain:

- H1: When decisions are private, people will choose sure gains (regardless of whether they are ethical or not).
- H2: When decisions are public, people will avoid choosing unethical options (regardless of whether they involve a probable or sure gain).
- H3: Unethical alternatives will be chosen less when decisions are public than when they are private (regardless of whether they involve a probable or sure gain).

Following the logic of Prospect Theory [2], we propose that when unethical decisions are guaranteed to remain in the private sphere, they are chosen when they involve sure profits (H1). We expect to find this result because we believe that the decision-making tendency of the certainty effect, along with the impossibility of being discovered and punished socially through disapproval from others, will make the unethical alternative more attractive, even though it might contradict one’s internal moral standards of behavior [30, 40, 41]. In other words, we believe that the aversion to risk will be greater than the socio-moral aversion to choosing unethical alternatives in a context of privacy. By contrast, taking into consideration the Sociometer Theory [26–30], we expect that in situations where people are informed that the decision will made public, they will choose the ethical alternatives to avoid the others’ disapproval (H2). This aversion for the unethical alternative is based on anticipated feelings of shame that arise when a decision that involves a moral transgression is exposed publicly [21, 23–25]. Therefore, in the case of a public situation, we expect that the socio-moral aversion will be greater than the aversion to risk. This assumption contradicts the Prospect Theory predictions, favoring approaches that give importance to the emotional charge implicit in the alternatives [6–8]. Finally, we expect to clarify whether knowing that our decisions are made public (transparent) is a determinant factor in avoiding choosing unethical alternatives (H3). From our point of view, the aversion produced by the risk of disapproval from others [30] and empirical results that associate high levels of transparency with lower levels of corruption [42], are sufficient evidence of the relevant role of transparency in reducing unethical decisions. In summary, we believe that making unethical choices transparent, will produce anticipated feelings of shame that will cause greater aversion than the mere act of making anonymous unethical decisions. This fact highlights the dissuasive value of transparency as a political measure designed to reduce unethical economic decisions.

## Method

### Participants

A total of 165 psychology students from a Spanish university, with ages ranging between 20 and 24 years old, participated in this study. Regarding gender, the sample included a total of 52 men (32%) and 113 women (68%). The experiment had four experimental conditions: 41 people were part of condition 1, 43 were in condition 2, 40 in condition 3, and 41 in condition 4.

### Design and instruments

The experimental design and instruments were quite similar to the ones used by Kahneman and Tversky [2] to determine both the risk percentages and the amounts chosen (rewards). In addition, an implicit emotional value was introduced in one of the decisions offered, specifically in the alternative that involves winning a certain amount of money through an unethical behavior (accepting the money from a grant you know has been awarded to you by mistake). The other alternative proposes winning a certain amount of money through an ethical behavior with a neutral emotional value [9, 10]. We chose selling photocopied material (student’s own notes, printed power-point presentations of the topics taught, etc.) as the ethical alternative because in the context of the local culture at this university, it is a common practice among the students. Both the University and the professors know about and support this practice because it allows students to obtain extra money that they can invest in new materials. In sum, it is a consolidated practice that does not pose an ethical dilemma for the members of this university community (it is viewed the same way as markets for second-hand goods). Thus, the distribution of the alternatives in the conditions was the following:

- In conditions 1 and 3 (first location): The unethical decision was made to coincide with alternative (a) or sure gain, and the neutral decision with alternative (b) or probable gain.
- In conditions 2 and 4 (second location): The neutral decision was made to coincide with alternative (a) or sure gain, and the unethical decision with alternative (b) or probable gain.

Additionally, as in the study by Da Costa, Andrade and Santos [13], the participants of the conditions 3 and 4 were informed that their decisions would be made public (public situation), and the participants of the conditions 1 and 2 were advised that their decision would remain anonymous (private situation). Therefore, in the private situation, the decisions that involved obtaining a profit through unethical behaviors were emotionally displayed with a socio-moral affective charge. However, in the public situation, the emotion of shame is expected to be triggered because the moral transgressions will be known by the public [25]. Summarizing, the study uses a 2 (Type of situation: private situation vs public situation) x 2 (Location of the unethical gain in the alternatives: first location vs second location) factorial design. Therefore, in the private situation, conclusions will be drawn about the socio-moral emotions of the unethical decision, whereas in the public situation the effect of the emotion of shame will be estimated.

In order to quantify the effect of the previous variables on the decision-making process, we considered both the percentage choosing alternative (a) compared to alternative (b), as well as the total percentage choosing unethical alternatives compared to ethical alternatives, in each situation. Next, the exact instructions provided to the participants in the four experimental conditions are presented.

The following text was used in the private situation (conditions 1 and 2): “Please, imagine that you have to choose between the two options offered below. Taking into account that your decision never will be known by anyone, mark your choice:” The following alternatives were offered depending on the condition:

*Condition 1*:

a) A sure win of 300 € from a grant that does not correspond to you.

b) An 80% chance of winning 400 € from selling photocopied material from previous courses, and the remaining 20% of not winning anything (because buyers may not pay you).

*Condition 2*:

a) A sure win of 300 € from selling photocopied material from previous courses.

b) An 80% chance of winning 400 € from a grant that does not correspond to you, and the remaining 20% of not winning anything (because the authorities finally find out and correct the mistake).

According to the prospective theory proposed by Kahneman and Tversky [2], in both conditions a greater proportion should choose the sure gain, coinciding with H1, which proposes that this will occur regardless of whether the choices leading to the sure gain are ethical or unethical. We consider that the socio-moral emotional charge that impregnates the unethical decision would not be strong enough to annul the decision tendency introduced by the aversion to risk because it is a private situation. In addition to the preference for sure gains introduced by the certainty effect, we have to add the fact that people are not running the risk of being discovered and/or punished by the disapproval of others [30, 40].

In the situation where the decisions are made public, the following text was offered: “Please, imagine that you have to choose between the two options offered below. Taking into account that your decision will be made public and known by everyone, mark your choice:” Again, the following alternatives were proposed depending on the condition:

*Condition 3*:

a) A sure win of 300 € from a grant that does not correspond to you.

b) An 80% chance of winning 400 € from selling photocopied material from previous courses, and the remaining 20% of not winning anything (because buyers may not pay you).

*Condition 4*:

a) A sure win of 300 € from selling photocopied material from previous courses.

b) An 80% chance of winning 400 € from a grant that does not correspond to you, and the remaining 20% of not winning anything (because the authorities finally find out and correct the mistake).

Analogically to what was stated about conditions 1 and 2, according to Prospect Theory [2], in conditions 3 and 4, the alternatives that guarantee a sure gain should be chosen to a greater extent. Nevertheless, based on proposals of the Sociometer Theory approach [26–30], we consider that people would avoid choosing those alternatives that could be the target of disapproval from others (H2). More specifically, the anticipated feelings of shame would make the unethical alternative less attractive, so that participants would avoid choosing it.

At the same time, we also expect that there would be significantly less avoidance of the unethical alternative in the private situation than in the public situation (H3) because the aversion introduced by the anticipated feeling of shame should be significantly greater than the one introduced by the socio-moral emotional charge. This assumption is based on transparency’s proven effect in reducing corruption [42], and on the aversion people have to public exposure of decisions that can put their relationship with others at risk [26–30]. In this regard, this difference in aversion will represent the degree to which transparency contributes to avoiding decisions that can result in unethical economic gains.

### Procedure

The university students (all of them over 18 years old) were asked to participate voluntarily in this experiment. Before entering the room where the experimental procedure was carried out, all of the students were informed of the conditions for participating in the experiment, and that they could stop responding to the written forms at any time they wished. Right before entering the room, they were asked if they wanted to continue with the experiment, in order to obtain their verbal consent. The privacy and anonymity of their answers were guaranteed at all times.

The experiment consisted of four experimental conditions randomly assigned to four independent groups of participants. Each group received a copy of one of the four versions of the decision-making problem described above. Participants were told that, after reading the statement attentively, they should choose one of the alternatives (or not answer, leaving the form blank if they did not want to participate in the study), and they were informed that there were no right or wrong answers.

In addition, approval was received from the Ethics Commission on Experimental Research at the University where the study took place. This commission is governed by the guidelines stipulated in the Helsinki Declaration.

### Data analysis

After calculating the frequency of the alternatives chosen by the participants, we performed the appropriate statistical analyses with the program SPSS 20. In order to confirm the proposed hypotheses, it was necessary to conduct both binomial and chi-squared (*χ*^2^) tests. The binomial tests were used to find out whether there were significant differences in the alternatives chosen in each experimental condition. The proportion applied to the binomial tests was 0.5. In addition, we used chi-squared (*χ*^2^) tests to carry out the proportion contrasts that would allow us to compare the percentages between different experimental conditions. These tests could determine whether there were statistically significant differences between conditions 1 and 2 (H1: private situation), between conditions 3 and 4 (H2: public situation), between conditions 1 and 3 and conditions 2 and 4 (H3: interaction effects), and between the percentages of choices in public and private situations (H3: main effects).

## Results

### Private situation

As Table 1 shows, in condition 1, the participants chose the alternative associated with probable gain to a greater degree, whereas in condition 2 the preferred alternative was the one linked to sure gain. These results indicate that in the private situation, the participants mainly avoided choosing the unethical alternatives, regardless of whether they involved sure or probable gains. To determine whether there were significant differences in the alternatives chosen in the two conditions, a chi-squared (*χ*^2^) test was performed, which compared the percentages of alternative (a) responses in each condition. The results suggest that there are statistically significant differences between condition 1 and condition 2 (*χ*^2^ = 30.527, p < .01), a result that contradicts the first hypothesis (H1) and implies that the socio-moral emotional charge that imbued the unethical choices in the private situation prevailed over the certainty effect proposed by Prospect Theory.

**Table 1:** Frequency and percentage in the private situation.

	Alternatives		N
	Sure gain	Probable gain	
**Condition 1**	16 (39%)^a^	25 (61%)^b^	41
**Condition 2**	41 (95%)^b^	2 (5%)^a^	43

^a^ Percentage of unethical gains

^b^ Percentage of ethical gains

In addition, to find out whether there were significant differences within conditions 1 and 2 in terms of preferences for sure or probably gains, a binomial test was carried out. Significant differences were found in condition 2 (p < .01), but not in condition 1 (p>.05), which indicates that the certainty effect was completely inverted in condition 2, but only suppressed in condition 1.

### Public situation

Table 2 show that in condition 3, people preferred probable gain, whereas in condition 4, they mainly chose sure gain. As in the private situation, most of the participants avoided choosing unethical alternatives, regardless of whether they involved sure or probable gain. These results are congruent with the second hypothesis (H2). In this regard, we used a chi-squared test (*χ*^2^) to compare the percentages choosing alternative (a) in each condition. The results suggest that there were statistically significant differences between conditions 3 and 4 (*χ*^2^ = 46.912, p < .01), and that, therefore, we can interpret that the unethical alternatives were avoided due to the anticipation of the emotion of shame, which was generated by the suggestion of making decisions public that would involve a transgression of a moral standard.

**Table 2:** Frequency and percentage in the public situation.

	Alternatives		N
	Sure gain	Probable gain	
**Condition 3**	8 (20%)^a^	32 (80%)^b^	40
**Condition 4**	39 (95%)^b^	2 (5%)^a^	41

^a^ Percentage of unethical gains

^b^ Percentage of ethical gains

The binomial tests revealed that there were significant differences in both condition 3 (p < .01) and condition 4 (p < .01). Specifically, the difference lies in avoiding the unethical choice in both conditions. Thus, the choice tendency implied by the certainty effect was completely inverted.

### Private situation versus public situation

To test the third hypothesis (H3), it was necessary to compare the main effect of the variable type of situation on the percentage of unethical choices made by participants, and consider the proportion of unethical choices stemming from the interaction between the two variables manipulated in the experiment (type of situation and location of the unethical decision in the alternatives). Table 3 reveal that fewer decisions were made involving unethical gain in the public situation (12%) than in the private situation (21%). However, with regard to the statistical analysis of the main effect, the results suggest that there were no significant differences between the private and public situations in avoiding the choice of unethical alternatives (*χ*^2^ = 1.711, p>.05).

**Table 3:** Frequency and percentage in the public and private situations.

	Alternatives		N
	Non-ethical gain	Ethical gain	
**Private situation**	18 (21%)^a^	66 (79%)^b^	84
**Public situation**	10 (12%)^b^	71 (88%)^a^	81

^a^ Percentage of sure gains

^b^ Percentage of probable gains

**Fig 1:**
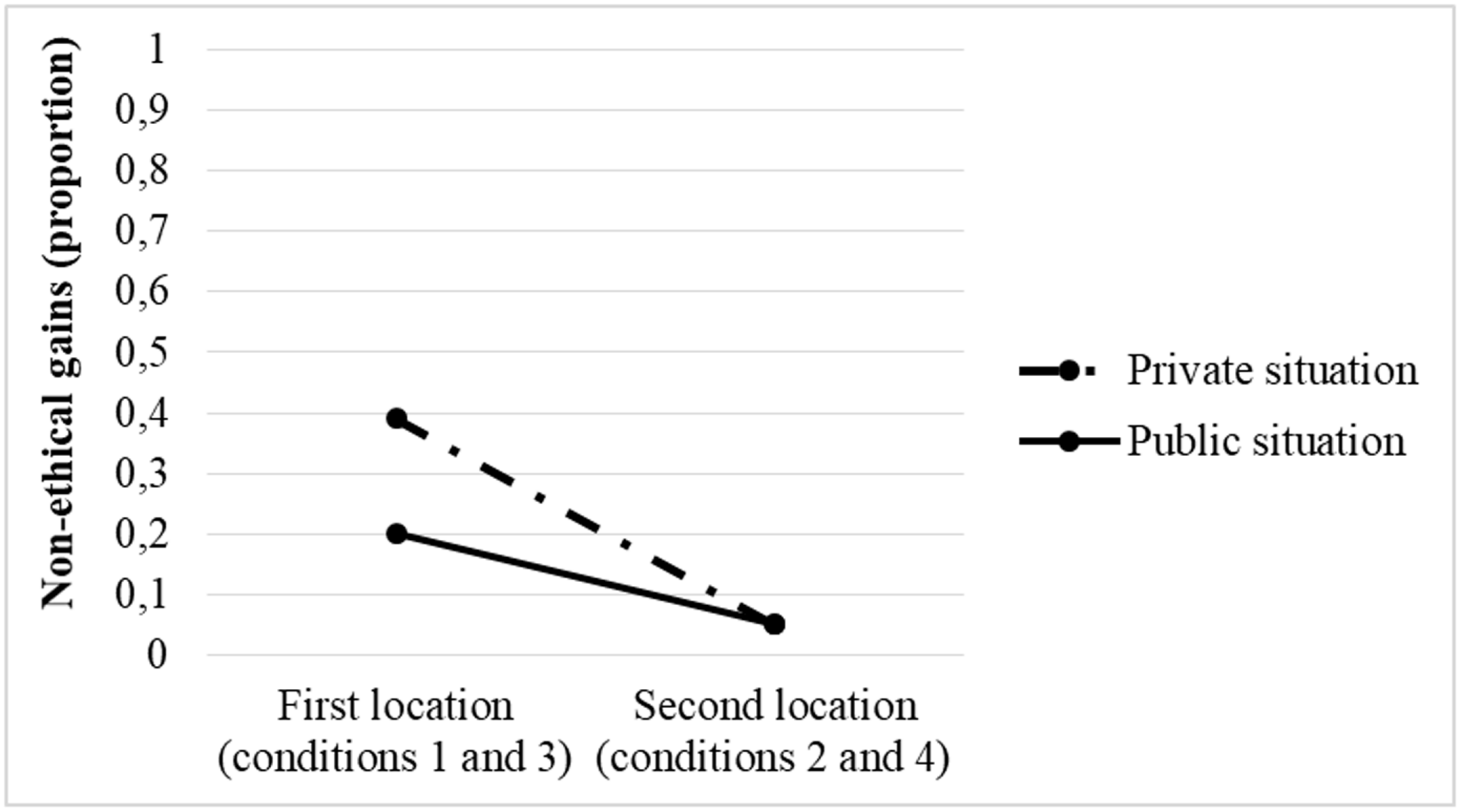
Locations of the unethical gain. Each line represents the percentage of unethical gains (from 0 to 1) chosen by the participants based on whether these gains are located in alternative (a) or (b) for private and public situations. In the first location (1), the unethical gain is associated with alternative (a), or sure gain, whereas in the second location (2), the unethical gain is associated with alternative (b), or probable gain.

By contrast, the analysis of the interactions did not reveal the same pattern of results. In the cases where the unethical choice is associated with a sure gain (conditions 1 and 3, see Fig 1), the results show statistically significant differences between decisions made in public situations or in private ones (*χ*^2^ = 3.515, p < .05). However, in the case where the unethical choice is associated with a probable gain (conditions 2 and 4), no significant differences were found between the two situations (*χ*^2^ = 0.002, p>.05).

## Discussion and conclusions

We have presented the reasons that the Expected Utility Theory and Prospect Theory proposals are fundamentally consequentialist and cognitivist. Both theories assign a main role to the evaluation of probability and the nature of the possible outcomes, relegating the emotional charge inherent to many choice alternatives to a background role. Other approaches such as the “risk as feeling hypothesis” [6], the “affect heuristic” [7] and the “somatic marker hypothesis” [8] point out that our decisions in risk situations depend on the reciprocal influence between the cognitive evaluation and the affective information. In this regard, different studies have tried to untangle the different effects that various discrete emotions have on decision-making [5]. However, socio-moral emotions such as shame have been studied very little in the area of decision-making in situations of risk [5]. Various experiments have shown that the anticipated feeling of shame leads to greater interpersonal cooperation in games that involve economic decision-making [35–37]. Moreover, it has been suggested that the emotion of shame can help to inhibit socially inappropriate behaviors involving a moral transgression [21, 30]. However, no experiment has tested the role that the anticipated emotion of shame plays in unethical decisions involving economic gain. For this reason, we proposed three hypotheses to clarify the role and effect of the anticipated emotion of shame on the area of decision-making in situations of risk. Additionally, we also considered that this influence can help to better understand the impact of transparency measures when trying to reduce unethical economic decision-making.

The hypothesis we propose about what occurs when decisions remain private (H1) is not supported by the results. In conditions 1 and 2, people preferred to avoid unethical alternatives rather than choosing an alternative that would guarantee sure gains. Consequently, the certainty effect proposed by the Prospect Theory was suppressed. This result is due to the fact that the participants in this experiment avoided the alternative they associated with a socio-moral emotional charge, which shows that aversion to the unethical alternative was greater than the aversion to risk. People were motivated to avoid making a decision that meant committing a transgression of an internal moral standard. Thus, we think the mechanism through which values are assigned to each alternative was altered, and less value was given to the decision that involved a gain obtained through unethical means, even though all the alternatives had an equivalent economic value. In addition, although it can be stated that there was risk-seeking in condition 2, it is not possible to state that there was risk-seeking in condition 1 because the binomial test did not verify this result.

However, H2, which corresponds to what would be expected when the situation is public, is confirmed by the results. In this case, the same response pattern is repeated as in the private situation, but with greater differences between conditions. This translates into a complete annulment of the certainty effect that would be predicted by Prospect Theory, again due to aversion to choosing an economic gain obtained through unethical means. Choosing the unethical alternative is associated with committing a moral transgression that will be publicly known by others, so that anticipated feelings of shame are high, which reduces the expected value of the unethical alternative. When anticipating the emotion of embarrassment, people avoid taking risks to keep others from viewing their bad actions [13], whereas when anticipating the emotion of shame, the key lies in avoiding morally inappropriate behaviors that can endanger one’s self-esteem and social inclusion [26].

Together, the results that affect the first two hypotheses show the fundamental weight of the socio-moral emotions in decision-making in situations of risk. More specifically, they show that the emotion of shame can take priority over other cognitive tendencies such as risk aversion. This is a relevant factor to take into account when estimating the decisions that people will make when they have to choose between various alternatives with similar economic values. Thus, whereas other studies show that being under a mental state of guilt is accompanied by an aversion to risk [14], anticipated feelings of shame lead to avoiding any decision that produces this emotional state, regardless of whether it is a risky decision or not.

H3 receives mixed support, given that its main effect is rejected, as well as the interaction when the unethical alternative is associated with a probable gain (conditions 2 and 4), but it is confirmed when the unethical alternatives coincide with sure gains (conditions 1 and 3). In general terms, this means that there were no significant differences in the avoidance of unethical alternatives when it was communicated that the decisions would be made public, compared to when they would remain anonymous. In other words, people avoided making unethical decisions to avoid a transgression of an internal moral standard, so that the corrective effect of transparence was relegated to a secondary role. Further exploring these results, when the unethical alternative was associated with a probable gain, there was no difference between making a decision in a public or private situation. This occurs because in conditions 2 and 4, the unethical alternative is doubly aversive, as it produces both socio-moral rejection (anticipated feeling of shame) and risk aversion (associated with a probable gain). In addition, the most interesting result is that (see Fig 1) when the unethical alternative was associated with a sure gain and, thus, would acquire a certain attraction compared to the emotionally neutral probable gain, the suggestion that the decisions would be made public had a more influential effect on avoiding mainly unethical alternatives. This result indicates that contradicting an internal moral standard is not always sufficient to guarantee that people will avoid making unethical decisions, and making the decision public, that is, transparent, can reduce moral infractions to the point of almost completely eliminating them. In this regard, socio-moral emotions such as shame can work as dissuasive mechanisms designed to reduce the incidence of unethical economic decisions.

In summary, although it seems true that people avoid making unethical decisions mainly to avoid a transgression of an internal moral standard, we cannot categorically state that transparence is useless in reducing unethical decisions. Perhaps it can be said that transparence has a moderate effect on determining the degree of integrity of our decisions. For this reason, approaches that seek to explain dishonesty, such as the theory of self-concept maintenance [41], should consider that people are also influenced by the existence of external socio-moral standards of behavior. This is important, especially taking into account that in developed countries, transparency measures co-exist with legislative codes and civic and moral education programs.

A limitation of the present study is that it only addresses the effects of the emotion of shame and the role of transparency in a hypothetical situation of gains. Other studies more focused on affect [9, 10] have also explored decision-making in a hypothetical situation of loss, or they have used low probabilities to determine the effects of associating emotional value on Prospect Theory predictions. Thus, future studies should establish the different functions of value that can arise by using different criteria, such as the type of discrete emotion produced when making a decision and the probabilities of success associated with each alternative. In addition, it would also be interesting to use different statements to check the degree to which transparency is effective in reducing unethical decisions about different types of behavior. It is quite likely that certain unethical behaviors receive very little rejection in certain societies, so that the dissuasive impact introduced by transparency measures might be, in the best case scenario, quite limited. In fact, we cannot rule out that in societies that are culturally permissive about breaking certain rules, not only would there be an annulment of the effect of the emotion of shame in reducing unethical decisions, but these immoral behaviors could even be reinforced by publicizing these infractions.

In conclusion, this study takes a first step toward understanding the psychological mechanisms underlying transparency measures as a means to reduce unethical economic decision-making. Thus, socio-moral emotions such as shame are keys to impeding moral transgressions, both in public and private settings. These results encourage us to consider new theoretical models that recover the importance of integrating the emotions in decisions made in risk situations, as only in this way can preventive measures to reduce unethical behaviors be more efficiently applied.

## Supporting information

S1 File Database description. Gender: male = 1; female = 2. Type of situation: private = 1; public = 2. Condition: condition = 1; condition 2 = 2; condition 3 = 3; Condition 4 = 4. Dependent variable: alternative (a) or sure gain = 1; alternative (b) or probable gain = 0.
(XLSX)
